# No Differences in Malnutrition Status and Prognostic Nutrition Index Between Female Patients with Systemic Lupus Erythematosus and Rheumatoid Arthritis: A Case-Control Study

**DOI:** 10.31138/mjr.051225.srq

**Published:** 2026-06-09

**Authors:** Arriana Gkouvi, Eleni C. Pardali, Sotirios G. Tsiogkas, Nektarios Marios Liapis, Christina G. Katsiari, Dimitrios P. Bogdanos, Maria G. Grammatikopoulou

**Affiliations:** 1 Immunonutrition Unit, Department of Rheumatology and Clinical Immunology, University General Hospital of Larissa, Faculty of Medicine, School of Health Sciences, University of Thessaly, Biopolis, Larissa, Greece;; 2 Ruhr-Universität Bochum, Germany and Rheumazentrum Ruhrgebiet, Herne, Germany

**Keywords:** disease activity, GLIM, malnourishment, rheumatology, underweight, weight loss

## Abstract

**Objectives:**

Malnutrition is increasingly recognised as a comorbidity in rheumatic diseases, but its clinical associations in systemic lupus erythematosus (SLE) and rheumatoid arthritis (RA) remain poorly described. The aim of this case-control study was to investigate malnutrition utilising the Global Leadership Initiative on Malnutrition (GLIM) criteria and the prognostic nutrition index (PNI) in patients with SLE and RA and explore potential associations with disease activity.

**Methods:**

In this case-control study, women with SLE (n=36) were compared to women with RA (n=36). Nutritional status was assessed using the GLIM criteria and the PNI was calculated. For the cases group (SLE), disease activity was measured using SLEDAI-2K. Associations between malnutrition and clinical variables were explored with multiple simple logistic regressions and a receiver operating characteristic (ROC) curve was produced to identify a cutoff value of the PNI that would better discriminate well-nourished from malnourished patients according to the GLIM.

**Results:**

Malnutrition was identified in both patients with SLE and RA, and only 8.3% of each group was classified as well-nourished. No differences were observed between SLE and RA concerning the nutritional status or PNI values. PNI was not associated with the SLEDAI-2K and ROC analysis showed limited discriminatory ability of PNI to identify GLIM-defined malnutrition (cut-off 8.44, sensitivity 0.73, specificity 0.50).

**Conclusions:**

Malnutrition constitutes an important issue in both SLE and RA. Routine nutritional assessment should be incorporated into clinical practice and management of the aforementioned conditions in order to ensure prompt identification and intervention.

## INTRODUCTION

Systemic lupus erythematosus (SLE) is a complex autoimmune rheumatic disease with variable manifestations that may affect nutritional status further complicating inflammatory response and disease prognosis. Although disturbances in appetite, dietary intake and body composition have been previously described in SLE,^1,2^ the extent to which these translate into a greater malnutrition burden relative to other rheumatic diseases remains less clearly defined. In SLE, fatigue, fever, pain (arthralgias, myalgias, headache), and loss of appetite may synergistically limit dietary intake, while neuropsychiatric and gastrointestinal manifestations may further alter dietary choices.^3,4^ As a result, research has revealed that patients with SLE tend to adopt unbalanced, micronutrient deficient diets, characterised by a low carbohydrate and fiber content and a high intake of protein and fat.^2,5,6^ In parallel, body weight loss consists of a common manifestation in SLE, experienced by 17– 51% of the patients^7^ mainly preceding disease diagnosis.^8^ Therefore, although many patients with SLE are overweight or obese,^9,10^ malnutrition is also common, affecting a great proportion of patients.^2,11^ According to recent research^12^ and as seen in other rheumatic diseases,^13,14^ weight loss is also associated with a reduction in fat-free mass and an increase in fat mass, leading to sarcopenia and cachexia. Furthermore, at some point in time, most patients tend to develop cachexia, with a great proportion of them never achieving body weight recovery.^12^

Low body mass index (BMI), steroid use, greater disease activity, vasculitis, lupus nephritis, serositis, and other factors seem to contribute to the observed weight loss in SLE.^11,12^ In SLE, malnutrition appears to be the synergistic result of pharmacotherapy, inflammation, disease manifestations and pain, all resulting in a reduced appetite and dietary intake. In parallel, various dietary changes have been shown to alleviate SLE symptoms,^15^ with patients frequently adopting elimination diets in an effort to tamper down symptomatology. Thus, the confluence of mineral and vitamin deficiencies and malnutrition interacts with the systemic presentation of nephritis, arthritis, vascular events and organ damage to the kidneys, heart, central nervous system, and skin, multiplying the morbidity and mortality of these patients.^16–19^ However, despite these important observations, it remains unclear whether the combined effects of reduced intake, disease manifestations and pharmacotherapy translate to a greater malnutrition risk in SLE. For all these factors, it is hypothesised that patients with SLE might be at greater malnutrition risk compared to other patients with rheumatic diseases. The present case-control study aimed to assess malnutrition in patients with an SLE diagnosis compared to controls (patients with rheumatoid arthritis [RA]).

## MATERIALS AND METHODS

### Patient recruitment

Female patients with an SLE diagnosis according to the 2019 European League Against Rheumatism (EULAR)/American College of Rheumatology (ACR) classification criteria,^20^ or a diagnosis of RA according to the ACR/EULAR 2010 criteria,^21^ were recruited from the Department of Rheumatology and Clinical Immunology, University Hospital of Larissa in central Greece, from December 2024 until March 2025. Approval for the study was granted from the Hospital’s Scientific Committee (Approval no. 30/3rd/20-02-2025) and informed consent was provided from all patients. The sample size was not calculated due to the fact that the data corresponded to a specific hospital in a defined region. Participant characteristics are presented in **Table 1**. Patients who met the following criteria were included in the study: (i) patients with an established diagnosis of SLE or RA (ii) able to communicate effortlessly in the Greek language (iii) female (iv) adults (v) that were willing to participate and provided informed consent. Individuals diagnosed with concomitant cancer, pregnant, with severe renal or hepatic failure, with current infection and those under the age of 18 were excluded from the study. All consecutive patients who met the inclusion criteria were recruited for the study.

**Table 1.** Patient characteristics (N=72).

	SLE (*n*=36)	RA (*n*=36)
Women (*n*)	36	36
Age (years)	49.4± 15.1†	63.8 ± 9.8†
	47 (20–77)^*^	64 (37–83)^*^
Diagnosis duration (years)	12.4 ± 8.2†	17.4 ± 15.0†
	11 (0.2–27)^*^	12 (1–57)^*^
BMI (kg/m^2^)	26.8 ± 6.24†	28.9 ± 5.47†
	25.7 (18.1, 45.6) ^*^	28.4 (19.4, 43.3)^*^
Weight status (*n, %*) (underweight/normoweight/over-weight-obese)	1 (2.8%) / 14 (38.9%) / 21 (58.3%)	0 (0%) / 8 (22.2%) / 28 (77.8%)
Caucasian/Roma (*n*)	35/1	36/0
Education (*n*) (primary/secondary/tertiary)	9/15/11	20/10/6

BMI: body mass index; RA: rheumatoid arthritis; SLE: systemic lupus erythematosus.

† Mean ± SD.

*: Median (min, max).

### Anthropometric indices

Body weight and height of participants were measured by an experienced dietitian (E.C.P.) using a digital floor scale (Kern MPE 200K-1PEM, Kern, Germany) and a wall stadiometer (Seca 220, Hamburg, Germany), respectively. BMI was calculated for all patients. Body weight status was defined according to the BMI cutoffs as underweight (< 18.5 kg/m^2^), normoweight (18.5 < BMI < 25 kg/m^2^), overweight (25 > BMI < 30 kg/m^2^), and obesity (BMI ≥ 30 kg/m^2^).

### Malnutrition assessment

One biomarker index and one composite score were used to assess malnutrition in the two groups.

The prognostic nutritional index (PNI) was calculated for each patient using the following equation: [(10 × serum albumin (g/dL)) + (0.005 × total lymphocyte count)].^22^ The PNI has been suggested as an easy-to-calculate bedside “malnutrition-inflammation” biomarker, with greater values indicating healthy nutritional status and lower values being indicative of malnutrition.

Malnutrition was also assessed using the Global Leadership Initiative on Malnutrition (GLIM) criteria.^23^ The GLIM criteria use a variety of phenotypic and etiologic criteria for the diagnosis of malnutrition including weight loss, reduced muscle mass, low BMI, reduced food intake or assimilation and inflammation.^23^ The diagnosis of malnutrition requires at least one phenotypic and one etiologic criterion.^23^

### Disease activity

For the cases group (patients with SLE), disease activity was assessed using the SLE Disease Activity Index 2000 (SLEDAI-2K).^24^ The SLEDAI-2K is based on 24 weighted items scored as present or absent. The index was calculated by experienced rheumatologists (N.L. and A.G.) and considers recent seizures, psychosis, organic brain syndrome, visual disturbance, new onset sensory or motor neuropathy, lupus headache, new onset stroke, vasculitis, arthritis, myositis, inflammatory-type rash, alopecia, Heme-granular or red blood cell (RBC) urinary casts, hematuria, proteinuria, pyuria, oral/nasal mucosal ulcers, pleuritic chest pain (with pleural rub/effusion/pleural thickening), pericarditis, high DNA binding, low complement, core temperature >38°C, platelets < 100 × 10^9^/L and white blood cell count (WBC) < 3 × 10^9^/L.^24^

### Statistical analyses

Continuous variables were calculated as means ± standard deviation (SD), while median and min/max are also reported. Normality was assessed using Shapiro-Wilk. Categorical data is reported as percentages, with the respective sample size (n). To assess potential associations between the GLIM categorisation and other variables, we reformed the data accordingly: patients were divided into two groups, the well-nourished ones (where each patient had a GLIM score of 0), and the malnourished one (containing patients that had received a GLIM score of 1 or 2). Multiple simple logistic regressions investigated the association between the aforementioned categorisation and PNI, years of disease diagnosis, body weight and diagnosis of SLE or RA. To convert the estimated coefficients to odds ratios (ORs), we used the exponential value of log (odds). We also performed a sub-group analysis including only patients with SLE to explore whether the mean SLEDAI differed between the malnourished and well-nourished patients based on GLIM scores. Finally, a receiver operating characteristic (ROC) curve was produced to identify a cut-off value of the PNI that would better discriminate well-nourished to malnourished patients according to the GLIM.

Analysis of variance was used to explore differences in GLIM categorisation between malnourished and well-nourished patients. Furthermore, the Mann-Whitney U test was employed to assess differences between scale variables and nutritional status. A *p* value of less than 0.05 was considered as statistically significant. All analyses were performed using R studio (version 4.4.1).^25^

## RESULTS

Median SLEDAI-2K values of patients with SLE was 2.00 (IQR: 2.75, min-max: 0–15). One fourth (25%) of the patient group with SLE had active disease (SLEDAI-2K ≥ 5). In the SLE arm, a small proportion (5.6%) of patients were severely malnourished according to the GLIM criteria compared to 8.3% of the patients with RA (**Table 2**). The majority of patients with lupus (86.1%) were moderately malnourished, similarly to the RA arm (83.3%). The remaining 8.3% of patients in each group were adequately nourished according to the GLIM criteria.

**Table 2.** Nutritional status of cases and controls (N=72).

Variables	SLE (*n*=36)	RA (*n*=36)	*p* value
Well-nourished* (*n*, %)	3 (8.3%)	3 (8.3%)	
Moderately malnourished* (*n*, %)	31 (86.1%)	30 (83.3%)	NS
Severely malnourished*	2 (5.6%)	3 (8.3%)	
PNI	11 (4.9)	10.6 (6.8)	NS

NS: not significant; PNI: prognostic nutrition index^22^; RA: rheumatoid arthritis; SLE: systemic lupus erythematosus.

* Based on the Global Leadership Initiative on Malnutrition criteria.^23^

In the analyses investigating associations between malnutrition status and other variables, no significant associations were observed between GLIM and duration of diagnosis, body weight, diagnosis and PNI (**Table 3**). Given the age difference between groups, the association between diagnosis and malnutrition status was evaluated using age-adjusted penalised logistic regression. Diagnosis group was not associated with malnutrition (adjusted OR for RA vs SLE =1.28, 95% CI 0.20–7.58; p=0.781) and age was also not associated with malnutrition (OR per 1-year increase=0.99, 95% CI 0.92–1.05).

**Table 3.** Results of univariate logistic regressions examining associations between the GLIM and related variables.

Variables	OR	95% CI	*p* value
Duration of diagnosis	0.99	0.93–1.06	0.790
Body weight	0.97	0.93–1.01	0.205
Diagnosis of SLE/RA	1	0.17–5.74	1
PNI	1.04	0.89–1.30	0.679

CI: Confidence intervals; GLIM: Global Leadership Initiative on Malnutrition criteria^23^; OR: Odds ratio; PNI: prognostic nutrition index^22^; RA: rheumatoid arthritis; SLE: systemic lupus erythematosus.

**Figure 1.**
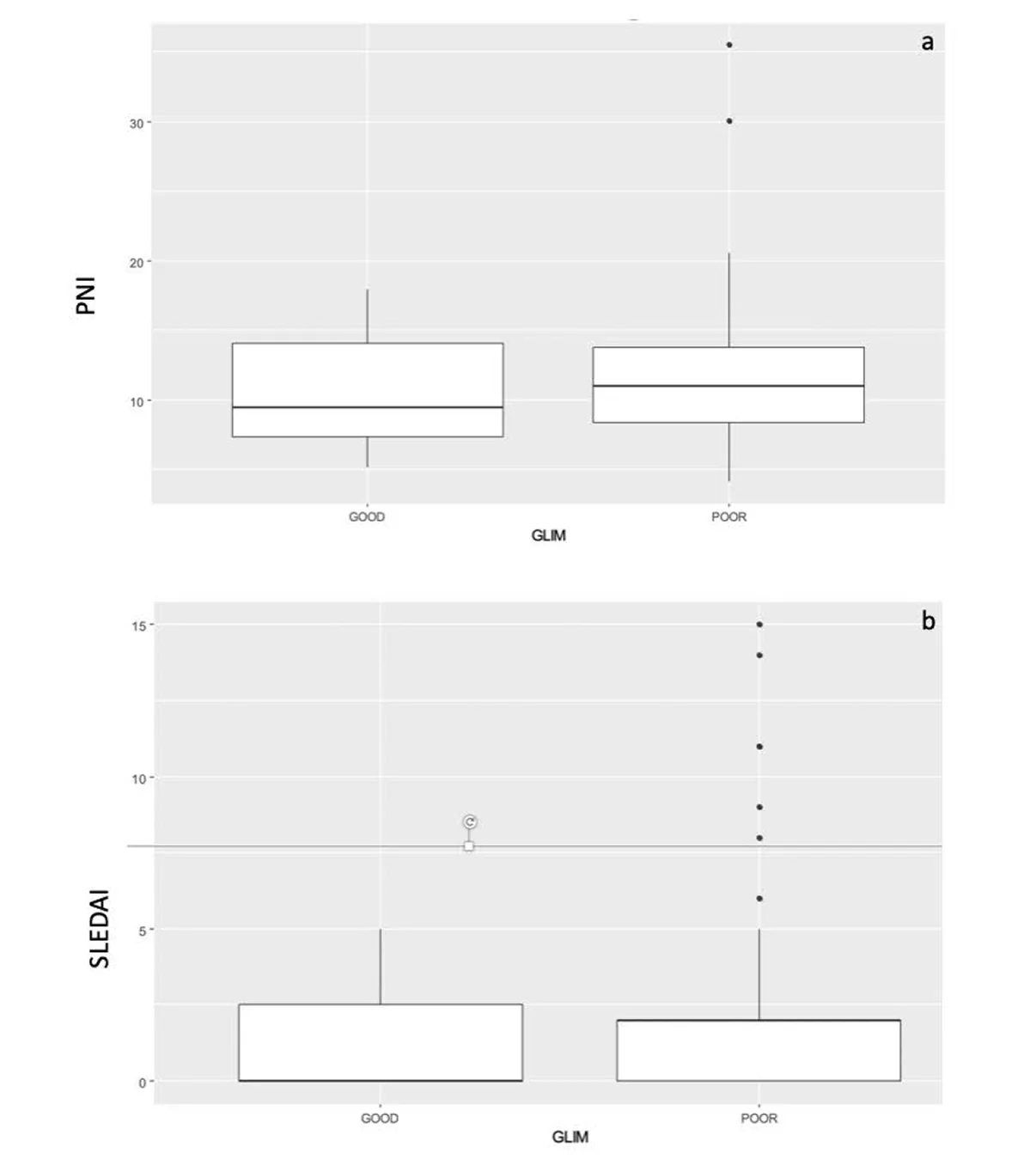
Differences in the PNI (a) and SLEDAI-2K (b) between malnourished and adequately nourished patients with SLE. PNI, prognostic nutrition index; SLE, systemic lupus erythematosus; SLEDAI, Systemic Lupus Erythematosus Disease Activity Index.

No difference was observed in the PNI or GLIM categorisation between patients with active or inactive lupus. In the sub-group analyses estimating differences in the SLEDAI and PNI between well-nourished and malnourished patients according to the GLIM, no differences were revealed (p=0.601 and p=0.685, respectively) (**Figure 1**).

**Figure 2.**
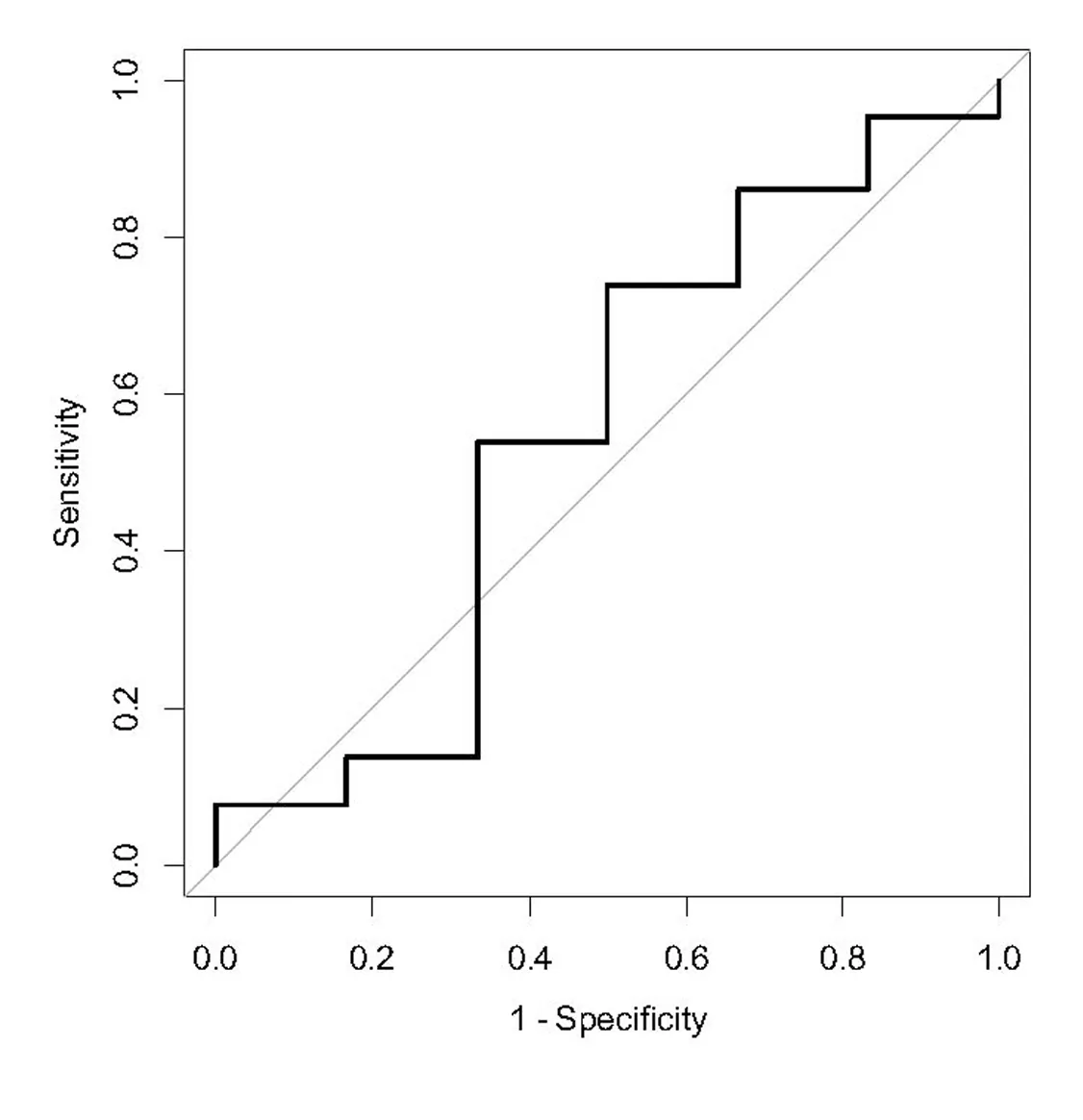
PNI ROC curve detecting threshold for patient discrimination according to malnutrition status. PNI, prognostic nutritional index; ROC, receiver operating characteristic.

**Figure 2** presents the ROC curve used to identify a cut-off value of the PNI that would better discriminate well-nourished versus malnourished patients according to the GLIM. The calculated threshold for PNI was 8.44, with a low specificity (0.5) and sensitivity (0.73). There were no missing data, apart from one person who did not disclose educational level.

## DISCUSSION

The present case-control study investigated the malnutrition status of patients with SLE and RA utilising the GLIM criteria and the PNI and further explored potential associations between malnutrition and disease activity. In our study sample, only 8.3% of the patients in each group were classified as well-nourished, while the remaining exhibited some degree of malnutrition. The high proportion of patients with malnutrition in RA when applying the GLIM criteria is consistent with previous reports, as in a cross-sectional study of women with RA, where malnutrition reached 71%.^26^ On the other hand, some studies using the same criteria have also reported lower malnutrition prevalence rates. Chen *et al.*^27^ performed a cross-sectional study on patients with autoimmune rheumatic diseases and reported that 26.5% and 22.9% of patients with SLE and RA respectively demonstrated malnutrition. It is important to note that evidence regarding malnutrition in SLE is still limited (**Table 4**). Borges et al.^2^ assessed malnutrition using another tool, the subjective global assessment (SGA), and report that only 8.2% of patients with SLE classified as malnourished. Interestingly, studies in systemic sclerosis revealed that the GLIM criteria tend to detect a higher prevalence of malnutrition compared to SGA.^28,29^ This difference reflects the underlying constructs of each tool: the SGA focuses on weight loss, gastrointestinal symptoms and reduced intake, while the GLIM further incorporates inflammation and muscle mass or strength.^23,30^ Supporting the relevance of muscle strength related parameters, a recent systematic review and meta-analysis noted that patients with SLE tend to have less muscle strength compared to healthy controls.^31^ Within the GLIM framework and taking into consideration additional contributors to myopenia in this population such as glucocorticoid use,^32^ patients with RA or SLE are therefore more likely to be classified as malnourished. In the present study this effect was particularly evident as only 18% of participants had normal handgrip strength and many fulfilled the inflammation criterion, due their underlying rheumatic disease diagnosis.

**Table 4.** Primary research on malnutrition among patients with SLE.

**Author**	**Origin**	**Design**	**Sample N**	**Malnutrition tools**	**Results**
Abou-Raya^41^	Egypt	CS	121 (98F/23M)	SGA, BMI	Most patients (43%) were overweight, 28.9% were obese, 10% were malnourished and 18.1% were normoweight. No SGA results were reported.
Ahn^33^	Korea	CS	217	PNI	Patients with active SLE exhibited lower PNI than those with inactive SLE. PNI increased as disease activity improved.
Behiry^43^	Egypt	CS	65	BMI	BMI revealed that more than 3/4 of patients were overweight and obese.
Borges^2^	Brazil	CS	170 (F)	SGA, BMI	Most (91.8%) patients were well nourished, 6.5% were moderately malnourished, and 1.8% were severely malnourished. In terms of BMI, malnutrition was diagnosed in 1.2% of the patients, 35.9% were normoweight, 35.3% were overweight, and the remaining 27.7% were obese.
Cohen^45^	Israel	CR	1	BW	The patient suffered from chronic abdominal pain and diarrhea, resulting to severe malnutrition, due to SLE-related protein losing enteropathy.
Correa-Rodríguez^11^	Spain	CS	173	NRI, PNI	Lower PNI and NRI scores were observed in active SLE patients than in inactive ones. PNI was inversely correlated with the SLEDAI and NRI positively correlated with SLEDAI and SDI scores. PNI and NRI were independent predictors of active SLE. Thus, both PNI and NRI may be useful markers to identify active SLE in clinical practice.
Huang^47^	USA	CR	1	BW	The patient exhibited a 3-year history of profound BW loss (300 lbs.), chronic diarrhea, recurrent small bowel obstruction and severe small bowel inflammation due to SLE-associated enteritis.
Lom-Orta^48^	Mexico	CSR	5	NR	All patients had severe PEM.
Meza-Meza^49^	Mexico	CS	130 (F)	BMI	Patients with excess body weight had a higher score of clinical activity and an association for high clinical activity, compared with non-overweight patients.
Pocovi-Gerardino^6^	Spain	CS	92	BMI	BMI was within normal range in 53.3% of patients, while 43.5% had excess BW.
Santos^50^	Brazil	CS	170 (F)	BMI	Two (1.2%) patients were underweight, 34.7% were normoweight, 35.9% were overweight, 28.3% were obese. Overweight and obesity were associated with older age, lower education, greater SLEDAI, higher complement concentration, hypertension, DM and ovarian failure, and less frequent antimalarial use.
Rabrenović^34^	Serbia	CC	67 (*n*=34 with active LN) and *n*=25 HC	PNI, CONUT, NRI	Between HC, patients with active and inactive LN, PNI and CONUT score differed. No differences were observed in the NRI between groups.
Sari^51^	Indonesia	CR	1	ASPEN criteria	The patient was severely malnourished, with a history of grade I obesity in the past 10 months prior to admission. She also experienced vitamin D deficiency and sarcopenia, with an FFMI of 13.6 kg/m^2^ and SMI of 2.5 kg/m^2^.
Stojan^12^	USA	Prospective cohort	2452	Cachexia based on the modified Fearon criteria	Within 5 years of cohort entry, 56% of the patients developed cachexia, with 18% never recovering their weight at follow-up. Risk factors for developing cachexia included a low BMI (<20 kg/m^2^), steroid use, vasculitis, LN, serositis, hematologic SLE manifestations, positive anti–dsDNA, anti-RNP and anti-Sm. Patients with intermittent cachexia had greater SDI scores compared to the rest.
Xia^53^	China	CS	240	BMI, albumin	9.5% were underweight, 49% normoweight, 22.1% overweight and 19.3% obese. Disease activity was an independant risk factor for malnutrition.

anti-dsDNA: anti–double-stranded DNA; anti-RNP: anti-ribonuclear protein; anti-Sm: anti-Smith; ASPEN: American Society for Parenteral and Enteral Nutrition; BMI: body mass index; BW: body weight; CC: Case control; CONUT: Controlling Nutritional Status; CR: case report; CS: cross-sectional; CSR: case series; DM: diabetes mellitus; F: female; FFMI: fat free mass index; HC: healthy controls; LN: lupus nephritis; M: male; NR: not reported; NRI: nutritional risk index; PEM, protein-energy malnutrition; PNI: prognostic nutritional index; SDI: Systemic Lupus International Collaborating Clinics/American College of Rheumatology Damage Index; SGA: subjective global assessment; SLE: systemic lupus erythematosus; SLEDAI: SLE disease activity index; SMI: skeletal muscle index.

Regarding the PNI, previous studies have suggested its potential importance as a proxy for inflammation and malnutrition status in the context of rheumatic diseases. In SLE specifically, the PNI has been reported to correlate with the SLEDAI in a mixed sex sample of patients.^11,33,34^ In RA, Öz *et al.*^35^ demonstrated that disease activity is inversely related to the PNI, while Isoda and associates^36^ showed that the PNI could aid the identification of older patients with RA, at risk of severe infections. Beyond RA and SLE, the PNI has also been linked to disease severity and relapse risk in anti-neutrophil cytoplasmic antibodies (ANCA) associated vasculitis^37^ and organ involvement in adult onset Still’s disease (AOSD).^38^ In contrast, the present study failed to identify significant associations between the PNI and SLEDAI-2K. Several factors may explain the lack of findings. First and foremost, the sample herein included only women, whereas men with SLE are more likely to develop organ damage such as renal involvement, neuropsychiatric SLE, cardiovascular or peripheral vascular disease.^39^ This could strengthen the association between the SLEDAI and PNI in mixed sex cohorts. Additionally, it is possible that the selected patients in the present study were mostly in remission, thus we lacked an adequate sample of patients with increased disease activity to verify this association. In fact, less than 1/3 of our sample had active disease (SLEDAI-2K ≥ 5), compared to the study by Ahn et al*.*^33^ where nearly half of the participants had active disease. In addition to the lack of correlation with disease activity, the ROC curve analysis also indicated that the PNI had limited value in identifying malnutrition in the present sample, with a cut-off of 8.44 yielding modest sensitivity (0.73) and low specificity (0.50).

Although the current study design treated patients with SLE as cases and patients with RA as controls, no meaningful differences emerged between these two groups, suggesting that malnutrition represents an equally important, shared burden among rheumatic disease diagnoses rather than being disease-specific. This finding highlights the need for systematic nutritional assessment in both conditions in order to prevent malnutrition and its detrimental complications. Multidisciplinary strategies are of great importance, including early referral to dietitians, implementation of tailored physiotherapy programs and pharmacological bone protection where appropriate.^40^

Limitations of the present study include the relatively modest sample size. Furthermore, the inclusion of only women limits generalisability and although participants could have been matched for disease duration, doing so would further restrict sample size. The age difference between the RA and SLE groups may have confounded between group comparisons. This was addressed using age-adjusted analyses, but residual confounding remains possible due to the small sample size. Finally, the low proportion of patients with active SLE may have reduced the ability to detect associations between disease activity and PNI. These results should therefore be interpreted as exploratory and require confirmation in larger, mixed sex cohorts with a wider distribution of disease activity.

## CONCLUSION

The present study showed that a mere 8.3% of patients with SLE are adequately nourished, while the remaining 91.7% are malnourished to some degree. These results necessitate frequent malnutrition screening and its integration in everyday clinical practice. Between SLE and RA, the risk for malnutrition appears to be similar.

## Data Availability

The data that support the findings of this study are available from the corresponding author upon reasonable request.
